# Comprehensive Determination of *Mycobacterium tuberculosis* and *Nontuberculous Mycobacteria* From Targeted Capture Sequencing

**DOI:** 10.3389/fcimb.2020.00449

**Published:** 2020-09-01

**Authors:** Ya He, Ziying Gong, Xiaokai Zhao, Daoyun Zhang, Zhongshun Zhang

**Affiliations:** ^1^Clinic and Research Center of Tuberculosis, Shanghai Key Lab of Tuberculosis, Shanghai Pulmonary Hospital, Tongji University School of Medicine, Shanghai, China; ^2^Shanghai Yunying Medical Technology Co., Ltd., Shanghai, China; ^3^Jiaxing Yunying Medical Inspection Co., Ltd., Jiaxing, China

**Keywords:** *Mycobacterium tuberculosis*, *nontuberculous mycobacteria*, diagnostic methods, drug resistance, targeted capture sequencing

## Abstract

Infection of *Mycobacterium tuberculosis* (MTB) and *nontuberculous mycobacteria* (NTM) challenges effective pulmonary infectious disease control. Current phenotypic and molecular assays could not comprehensively and accurately diagnose MTB, NTM, and drug resistance. Next-generation sequencing allows an “all-in-one” approach providing results on expected drug susceptibility testing (DST) and the genotype of NTM strains. In this study, targeted capture sequencing was used to analyze the genetic backgrounds of 4 MTB strains and 32 NTM pathogenic strains in 30 clinical samples, including 14 sputum specimens and 16 bronchoalveolar lavage fluid samples. Through comparing with other TB diagnostic tests, we proved that targeted capture sequencing could be used as a highly sensitive (91.3%) and accurate (83.3%) method to diagnose TB, as well as MGIT 960. Also, we identified 7 NTM strains in 11 patients; among them, seven patients were MTB/NTM co-affected, which indicated that it was a meaningful tool for the diagnosis and treatment of NTM infection diseases in clinic. However, based on a drug-resistant mutation library (1,325 drug resistance loci), only 9 drug resistance strains and 22 drug resistance loci were discovered, having considerable discordance with the drug-resistant results of MGIT 960. Our finding indicated that targeted capture sequencing approach was applicable for the comprehensive and accurate diagnosis of MTB and NTM. However, from data presented here, the DST results identified by next-generation sequencing (NGS) showed a relatively low consistency with MGIT 960, especially in sputum samples. Further work should be done to explore the reasons for low drug-resistance detection rate of NGS.

## Introduction

Tuberculosis, a largely infectious disease caused by *Mycobacterium tuberculosis* (MTB) infection, remains a major global health threat with an estimated 10.4 million new cases and 1.8 million deaths in 2015 alone (World Health Organization, 2016). In recent decades, attributable to the rising incidence of HIV infection and the significant improvement in the laboratory techniques and diagnostic methods, *nontuberculous mycobacteria* (NTM) infections have also experienced an increasing incidence worldwide (Marras and Daley, 2002; Griffith et al., 2003). Also, it is now proved that various types of NTM also can affect humans and may cause either symptomatic or asymptomatic infection (Baghaei et al., 2012). Resistance has been reported to all drugs that are used to treat tuberculosis and NTM diseases, especially those that are multidrug resistant (MDR) and extensively drug resistant (XDR) (Xu et al., 2014; Dheda et al., 2017). Increased resistance is associated with decreased patient survival and is a substantial threat to disease control. According to WHO's report, the treatment success rates was 52% for MDR-TB and 28% for XDR-TB (World Health Organization, 2016).

The emergence and outbreak of MTB and NTM infections, especially MDR and XDR strains, have created a need for improved detection tools to guide treatment options for patients. Traditional phenotypic assays, such as Acid-fast Bacilli and LJ culture, could not distinguish between MTB and NTM, and the sensitivity (30–60%) is not high enough to satisfy clinical needs (Rath et al., 2013; Verma et al., 2013). As the gold standard, BACTEC MGIT 960 system has the ability to distinguish between MTB and NTM, but the NTM strains identified may not be a human pathogenic NTM (Hasan et al., 2013). Molecular-based diagnosis and drug susceptibility testing (DST) methods are more rapid and microbiologically safe compared with phenotypic assays. However, rapid molecular assays are currently limited for drug susceptibility test, as Xpert MTB/RIF, Line Probe Assays, or other DST assays perform inadequately for anti-TB drugs for the limited number of loci they examine (Coll et al., 2015).

Advances in next-generation sequencing (NGS) technology have expanded opportunities for genome analysis in the clinical laboratory. The NGS approach allows an “all-in-one” approach providing results on expected sensitivity of the strains, genetic background, epidemiological data, and indication of risk of laboratory cross-contamination (Cirillo et al., 2016). NGS is also an affordable method for genotyping hundreds of MTB isolates (Walker et al., 2013). However, NGS from the direct diagnostic specimen is not yet standardized, and to date, the most promising approaches are whole genome sequencing (WGS) from early positive liquid culture (Pankhurst et al., 2016). WGS has been applied in a clinical setting for the potential to overcome such problems and extend rapid testing to the full range of anti-TB drugs (Nimmo et al., 2017, 2019; Doyle et al., 2018). However, data complexity, human host background interference and expensive cost have restricted their clinical application.

In this study, we designed specific probes to capture MTB and NTM phylogenetic single nucleotide polymorphisms (SNPs) and 1,325 drug resistance markers based on a reported TB mutation library (Coll et al., 2015). Illumina NGS was used to comprehensively diagnose MTB and NTM, as well as MDR strains and XDR strains. To further assess potential benefits of the targeted capture sequencing, we compared our results with other traditional diagnostic and DST approaches, like Acid-fast Bacilli, LJ culture, TB-DNA, TB-RNA, T-SPOT, and BACTEC MGIT 960 system.

## Materials and Methods

### Patients

Our study was approved by the Ethics Committee of Shanghai Pulmonary Hospital (protocol no. 201709002) and was performed in accordance with the Declaration of Helsinki with regard to ethical principles for research involving human subjects. Written informed consent was obtained from all of the patients before participation in our study.

Between September 5, 2016 and April 15, 2017, we prospectively enrolled consecutive individuals with suspected pulmonary tuberculosis or NTM infection in Shanghai Pulmonary Hospital. All patients who were suspected of having active TB were tested using the Acid-fast Bacilli sputum smear, LJ culture tests, TB-DNA assay [Mycobacterium Tuberculosis DNA Fluorescence Diagnostic Kit (PCR-Fluorescence Probing); Sansure Biotech, Hunan, China], TB-RNA assay (TB-SAT, Biorise Group, Shanghai, China), and BACTEC MGIT 960 system at enrollment, in addition to the T-SPOT. Among these methods, the result of MGIT 960 test was used as the gold standard.

Susceptibility testing for the first-line anti-TB drugs rifampicin (RMP), isoniazid (INH), ethambutol (EMB), and streptomycin (STR) and the second-line drugs amikacin (AMK), capreomycin (CAP), and ofloxacin (OFX) was performed on all samples with the MGIT 960 system (Becton Dickinson, NJ, USA), according to the manufacturer's instructions.

### Sample Collection and DNA Extraction

A total of 30 patients were enrolled, including 14 sputum specimens and 16 bronchoalveolar lavage fluid (BALF) specimens. Sputum samples were collected using clean, dry, and leak-proof sputum cups. Samples were immediately placed in a cold box and transported to Shanghai Yunying Medical Technology Co., Ltd. (Shanghai, China).

DNA was specifically separated from the other cellular components with a high salt hexadecyl trimethyl ammonium bromide extraction. DNA was then extracted with a chloroform/isoamyl alcohol method and quantified using fluorometric quantification, Qubit 3.0 Fluorometer with a dsDNA Broad Range Assay Kit (Thermo Fisher Scientific, MA, USA).

### Library Preparation and Sequencing

At first, GenBank (.gb) files of 4 MTB strains and 32 NTM pathogenic strains were acquired (Supplemental Table 1: https://figshare.com/s/aac8b59d0fcc3c5ca745). We converted these GenBank (.gb) files into fasta files. All these reference sequences were merged to compose a multifasta reference sequence.

According to the previous report, mutation a library that comprised 1,325 polymorphisms (SNPs and indels) at 992 nucleotide positions from 31 loci, 6 promoters, and 25 coding regions was constructed (Coll et al., 2015). RNA oligonucleotide baits of 120-bp length spanning the total length of 3 kb in the non-repetitive region of each MTB and NTM strains was designed using an in-house Perl script developed by the PATHSEEK consortium. The specificity of the baits was verified by BLASTn searches against the Human Genomic + Transcript database. The custom-designed MTB and NTM bait library was uploaded to SureDesign and synthesized by Agilent Technologies.

Before processing DNA samples were quantified and carrier human genomic DNA (Promega) was added to obtain a total of 3 μg DNA input for library preparation. All DNA samples were sheared to an average size of 300 bp by ultrasonication (Covaris S220). The samples were then subjected to library preparation using the VAHTS Universal DNA Library Prep Kit for Illumina Paired-End Sequencing Library protocol (V1.4.1 Sept 2012). DNA (500 ng) was included in each hybridation (24 h, 65°C). The resulting library was run on a Nextseq 500 (Illumina, CA, USA).

### Bioinformatic Pipeline

**Figure 1:**
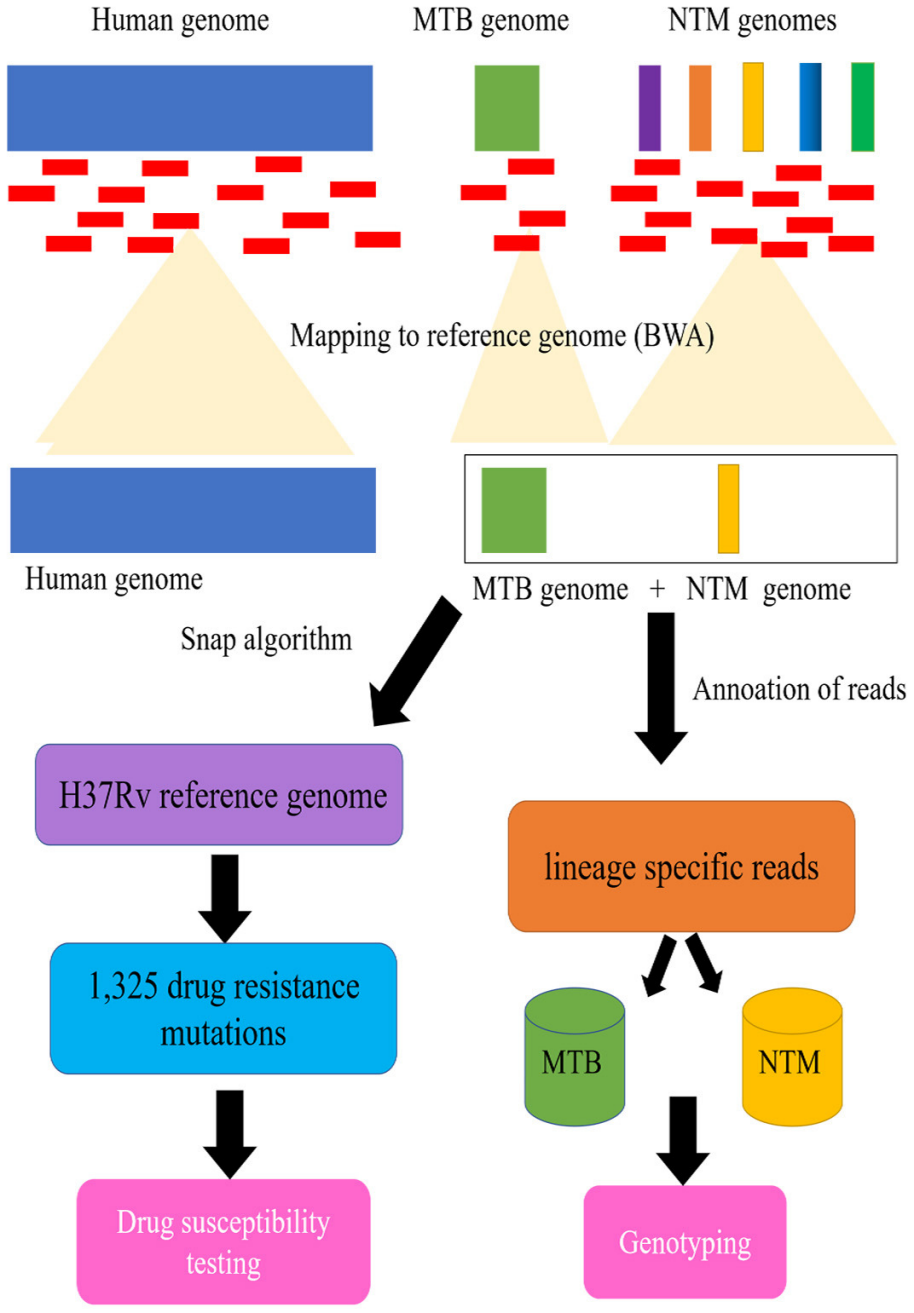
Schematic flow diagram illustrating processing of clinical samples for drug susceptibility testing (DST), and genotyping of mycobacteria belonging to *Mycobacterium tuberculosis complex* (MTB) and *nontuberculous mycobacteria* (NTM).

As seen in Figure 1, a pipeline was constructed for processing of clinical samples for DST, and genotyping of mycobacteria belonging to MTB and NTM species. First, as mentioned earlier, a multifasta reference sequence containing 4 MTB strains and 32 NTM pathogenic strains were acquired. Apart from this, to annotate the MTB/NTM gene, we also parsed the GenBank (.gb) files to generate a MTB/NTM gene reference having nucleotide intervals for each gene of each MTB/NTM type.

Second, we indexed the multifasta reference file using BWA aligner followed by alignment of reads to indexed genome. The aligned reads were extracted from the same file using a utility ViewSam from Picard Tools package (http://broadinstitute.github.io/picard/). For MTB/NTM type detection, the alignment files were parsed using UNIX shell program to detect the type of MTB/NTM as well as number of reads that align to a particular MTB/NTM type.

For the DST detection of patients with MTB infection, we map the aligned reads to the modified H37Rv reference genome (GenBank accession no. NC_000962.3), which consists of the genes and flanking regional sequences containing the selected 1,325 drug resistance mutations (Supplemental Table 2 https://figshare.com/s/1edc881504aeb196037a). The genome coverage of each sample was generated with Qualimap v2.2.1 (Okonechnikov et al., 2015). As previously described, the Snap algorithm (Zaharia et al., 2011) was used to rapidly characterize the mutations from the sequencing files (fastq format), and call SNPs and indels using samtool/vcf tools of high quality (Q30, 1 error per 1000 bp) (Coll et al., 2014b). All high-quality SNPs and indels identified from the alignments are compared with the curated list to determine known and novel polymorphism. Algorithmic results obtained were compared with standard SNP calling procedures using the full reference genome (Coll et al., 2014a).

### Statistical Analysis

Statistical calculations were made using Microsoft Excel (Redmond, Washington, USA) and SPSS 19.0 statistical package (SPSS, Chicago, Illinois, USA). A *p* < 0.05 was considered to be statistically significant. Coincidence rates between different detection platforms were calculated by using Cohen's kappa. Among these methods, the result of MGIT 960 test was used as the gold standard for the sensitivity, specificity, and accuracy measurements.

## Results

### The Diagnosis Results of Targeted Capture Sequencing

**Figure 2:**
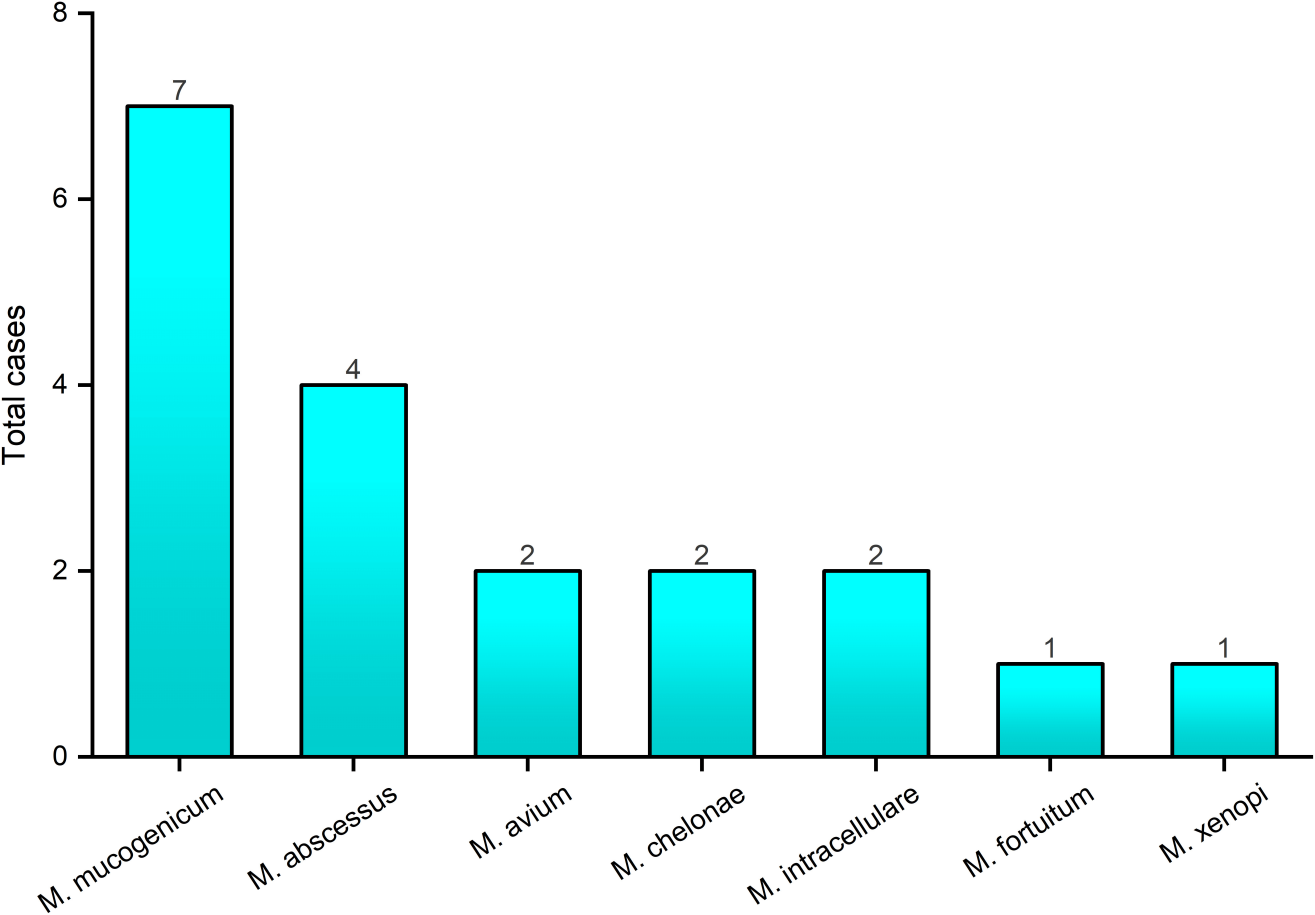
The genotyping results of *nontuberculous mycobacteria* by targeted capture sequencing. The *x*-axis represents the *nontuberculous mycobacteria* strains identified by targeted capture sequencing; the *y*-axis represents the number of cases with *nontuberculous mycobacteria* infection.

In total, 30 patients were enrolled in the study, including 12 men and 18 women, and the average age (SD) of the patients was 53.8, ranged from 20 to 81 (Table 1). DNA extracted from 14 sputum and 16 BALF specimens was sequenced. Among the 30 patients, targeted capture sequencing results showed 25 (83.3%) patients were positive for MTB and NTM. Seventeen (56.7%) cases were found to be affected by MTB, 15 (88.2%) of these cases were men. The mean age (SD) of MTB affected patients was 48.7 (±18.7). Otherwise, 15 (50%) cases were diagnosed to be affected by NTM. Nine (60%) of these cases were women and the mean age (SD) of the patients was 54.2 (±18.1). Seven cases (46.7%) were found to have MTB/NTM co-affected, including three cases which were affected by two or three NTM strains, simultaneously (Table 1). As shown in Figure 2, seven human pathogenic NTM strains were determined in this research. *Mycobacterium mucogenicum* and *M. abscessus* were discovered in seven and four patients, respectively, more than other NTM strains. All patients were negative for HIV except one patient, which was affected by *M. mucogenicum*. The detailed sequencing results are shown in Supplemental Table 3 (https://figshare.com/s/06e1c3b2c38fc0a4a648).

**Table 1:** The clinical information and targeted capture sequencing results of 30 enrolled patients.

**Characteristic**	**Number of patients**	**MTB/NTM infected**		
		**MTB infected**	**NTM infected**	**MTB/NTM co-affected**
Total	30 (100%)	17 (56.7%)	15 (50%)	7 (23%)
Gender				
Male	12 (40%)	15 (50%)	6 (20%)	6 (20%)
Female	18 (60%)	2 (6.7%)	9 (30%)	1 (3.3%)
Age (20–81)				
≤60	16 (53.3%)	11 (36.7%)	8 (26.7%)	3 (10%)
>60	14 (46.7%)	6 (20%)	7 (23.3%)	4 (13.3%)
HIV infected	1 (3.3%)	–	1 (3.3%)	–

*MTB, Mycobacterium tuberculosis complex; NTM, nontuberculous mycobacteria*.

### Comparison With Other Diagnosis Methods

**Figure 3:**
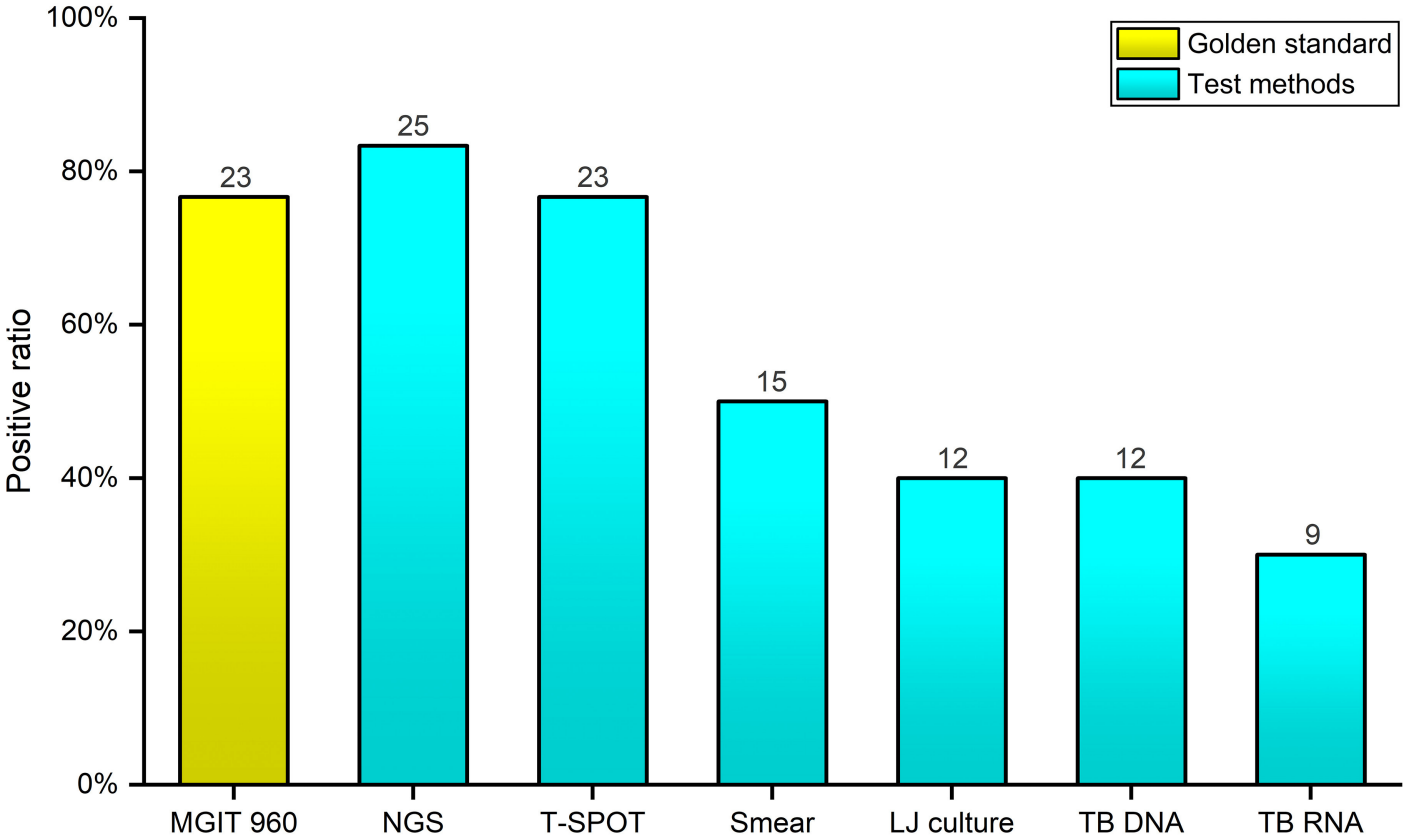
Comparison of the positive rates of seven diagnostic methods for *Mycobacterium tuberculosis complex* and *nontuberculous mycobacteria*. The *x*-axis represents the seven different diagnostic methods; the *y*-axis represents the positive rate of *Mycobacterium tuberculosis complex* and *nontuberculous mycobacteria* identified by each method.

Out of 30 enrolled samples, 15 (50%) were smear positive; LJ culture yielded 12 (40%) growths; positive results for TB-DNA and TB-RNA were found in 12 cases (40%) and 9 cases (30%), respectively; for T-SPOT. *TB* test, 23 cases (76.7%) were positive and 7 cases (33.3%) were negative. As the gold standard, BACTEC MGIT 960 system found that 23 (76.7%) samples presented positive results, and 7 (23.3%) samples presented negative results. Overall, NGS detected 25 (83.3%) positive patients, having a highest positive rate (Figure 3). For each patient, the detail diagnosis results of seven approaches are exhibited in Supplemental Table 4 (https://figshare.com/s/25cad926a4436c5998ba).

Comparing with the BACTEC MGIT 960 system, the sensitivity, specificity, and accuracy of various TB diagnostic approaches were calculated by SPSS 19.0, separately (Table 2). For Acid-fast Bacilli sputum smear, TB were not identified in eight individuals (65.2% sensitivity), whereas the specificity was high (100% specificity). Only 12 cases were detected by LJ culture (52.1% sensitivity), but the specificity of LJ culture was also high (100% specificity). The sensitivity and specificity of TB-DNA was 34.8 and 42.9%, having a worst accuracy (36.7%). The identified results of TB-RNA test also presented a lower consistency (30.4% sensitivity, 71.4% specificity, and 40% accuracy). As an accurate and sensitive *in vitro* diagnostic test, T-SPOT. *TB* had a higher sensitivity (78.3%) than other conventional tests; however, the lowest specificity (28.6%) was found in this study. It is worth noting that the sensitivity and accuracy of NGS were both highest (91.3% sensitivity, 83.3% accuracy) among these tests.

**Table 2:** The sensitivity, specificity, and accuracy of six diagnostic methods for *Mycobacterium tuberculosis complex* and *nontuberculous mycobacteria* compared with BACTEC MGIT 960 system.

**Methods**	**Sensitivity (95% CI)**	**Specificity (95% CI)**	**Accuracy (95% CI)**
AFB	65.2 (42.8–82.8)	100 (56.1–100)	73.3 (71.8–74.8)
LJ culture	52.1 (31.1–72.6)	100 (56.1–100)	63.3 (61.8–64.8)
TB-DNA	34.8 (17.2–57.2)	42.9 (11.8–79.8)	36.7 (34.2–39.2)
TB-RNA	30.4 (14.1−53.0)	71.4 (30.3–94.9)	40 (37.5–42.5)
T-SPOT	78.3 (55.8–91.7)	28.6 (5.1–69.7)	66.7 (65.3–68.2)
Targeted capture sequencing	91.3 (70.5–98.5)	42.9 (11.8–79.8)	83.3(82.3–84.3)

We also compared the diagnosis results of targeted capture sequencing with MGIT 960 system, entirely. We found that the positive cases of MTB and NTM were both more than the culture results of MGIT 960 (MTB: 16 vs. 12; NTM: 15 vs. 11). Seven patients (23.3%) were identified MTB/NTM co-affected by targeted capture sequencing; however, no one was discovered by MGIT 960 system (Table 3).

**Table 3:** Comparison of the positives rates of *Mycobacterium tuberculosis complex, nontuberculous mycobacteria*, and co-infection diagnosed by targeted capture sequencing and BACTEC MGIT 960.

**Methods**	**MTB**	**NTM**	**MTB + NTM**
BACTEC MGIT 960	12 (40%)	11 (36.7%)	–
Targeted capture sequencing	16 (53.3%)	15 (50%)	7 (23.3%)

*MTB, Mycobacterium tuberculosis complex; NTM, nontuberculous mycobacteria*.

### DST Using Bactec MGIT 960 System and NGS

For the 17 MTB-infected patients identified by targeted capture sequencing, MGIT 960 system was performed to detect the DST of RMP, INH, EMB, SRT, CAP, OFX, and AMK. Among the 17 TB patients, 82.4% (14/17) cases were identified resistant to at least two of the tested drugs, and 52.9% (9/17) cases were resistant to all the seven tested drugs. Four cases (23.5%) were found to be resistant to four drugs; case 28 and case 30 were resistant to five and six drugs, respectively (Table 4).

**Table 4:** Comparison of the drug susceptibility testing results of BACTEC MGIT 960 and targeted capture sequencing.

**Sample number**	**BACTEC MGIT 960**	**Targeted capture sequencing**
1	STR, INH, RMP, EMB, CAP, AMK, OFX	–
2	STR, INH, RMP, EMB, CAP, AMK, OFX	CAP/STR/AMK/KAN (rrs:1473329); CAP/AMK/KAN (rrs:1473247)
3	STR, INH, RMP, EMB, CAP, AMK, OFX	–
4	STR, INH, RMP, EMB, CAP, AMK, OFX	CAP/AMK/KAN (rrs:1473247), CAP/STR/AMK/KAN (rrs:1473329)
5	STR, INH, RMP, EMB, CAP, AMK, OFX	–
6	STR, INH, RMP, EMB, CAP, AMK, OFX	INH (katG:2154724), EMB (embB:4248115)
7	STR, INH, RMP, EMB, CAP, AMK, OFX	–
8	STR, INH, RMP, CAP, AMK	–
9	STR, INH, RMP, CAP, AMK, OFX	–
10	–	–
11	–	–
12	STR, INH, RMP, EMB, CAP, AMK, OFX	–
13	–	–
14	–	–
15	STR, INH, RMP, EMB, CAP, AMK, OFX	CAP/AMK/KAN (rrs:1473247), CAP/STR/AMK/KAN (rrs:1473329)
16	–	
17	STR, INH, RMP, EMB, CAP, AMK, OFX	FLQ (gyrA:9304), RMP (rpoB:761155;763031), AMK/KAN (rrs:1473246), STR(rpsL:781687), PZA (pncA:2289016, rpsA:1834177), INH (katG:2155168), EMB (embC:4242643, embA:4243460, embB:4247429)
18	–	–
19	STR, INH, RMP, EMB	STR (rrs:1472307), CAP/KAN/AMK (rrs:1473247), CAP/KAN/AMK/STR (rrs:1473329)
20	STR, INH, RMP	–
21	STR, INH, RMP, EMB, CAP, AMK, OFX	FLQ (gyrA:9304), CAP/AMK/KAN (rrs:1473247)
22	STR, INH, RMP, EMB, CAP, AMK, OFX	–
23	STR, INH, RMP, EMB, CAP, AMK, OFX	–
24	INH, RMP	–
25	INH, EMB, CAP, AMK	STR (rrs:1472644)
26	–	FLQ (gyrA:7585;9304), RMP (rpoB:763031), STR (rpsL:781822) PZA (rpsA: 183417)
27	STR, INH, RMP, OFX	–
28	STR, INH, CAP, AMK, OFX	–
29	–	–
30	STR, INH, RMP, CAP, AMK, OFX	–

However, by mapping to the mutation library, targeted capture sequencing only identified nine clinical samples with resisting to anti-TB drugs, including STR, KAN, EMB, INH, RMP, FLQ, CAP, PZA, and AMK. Among the nine patients, eight were resistant to at least two of the anti-TB drugs, which was mostly consistent with the culture results of MGIT 960 system. However, one patient was DST negative for MGIT 960 culturing and showed resistance to FLQ, RMP, STR, and FZA (Table 4). Cases 18 and 29 were detected with no drug resistance mutation, and the results of the analysis accorded with the MGIT 960 culturing ones. Mutations of the *katG, embB, embA, embC, rrs, gyrA, rpsL, rpsA, pncA*, and *rpoB* genes were identified by NGS shown in Table 4. Two INH-resistant clinical samples (case 6 and case 17) had a mutation in *katG* gene with two loci (2,154,724 and 2,155,168). Also, these two clinical samples were found to be resistant to EMB; one had a mutation in *embB* (loci: 4,248,115) and another one had mutations in *embA* (loci: 4,243,460), *embB* (loci: 4,247,429), and *embC* (loci: 4,242,643) genes. Seven STR-resistant clinical samples had mutations in *rrs* gene (loci: 1,472,644, 1,473,247, and 1,472,307), and in *rpsL* gene (loci: 781,822 and 781,687), respectively. Two FLQs-resistant clinical samples all had a mutation in *gyrA* gene (loci: 9,304 and 7,585). Four patients who had a mutation at the 1,473,247 loci and 1,473,329 loci in *rrs* gene are resistant to CAP/KAN/AMK/STR drugs, simultaneously. Otherwise, mutations of *gyrA, rpoB, rpsL, pncA, rpsA, katG, embB, embA*, and *embC* were all detected in one patient, which may be resistant to FLQ/RMP/STR/PZA/INH/EMB drugs.

**Figure 4:**
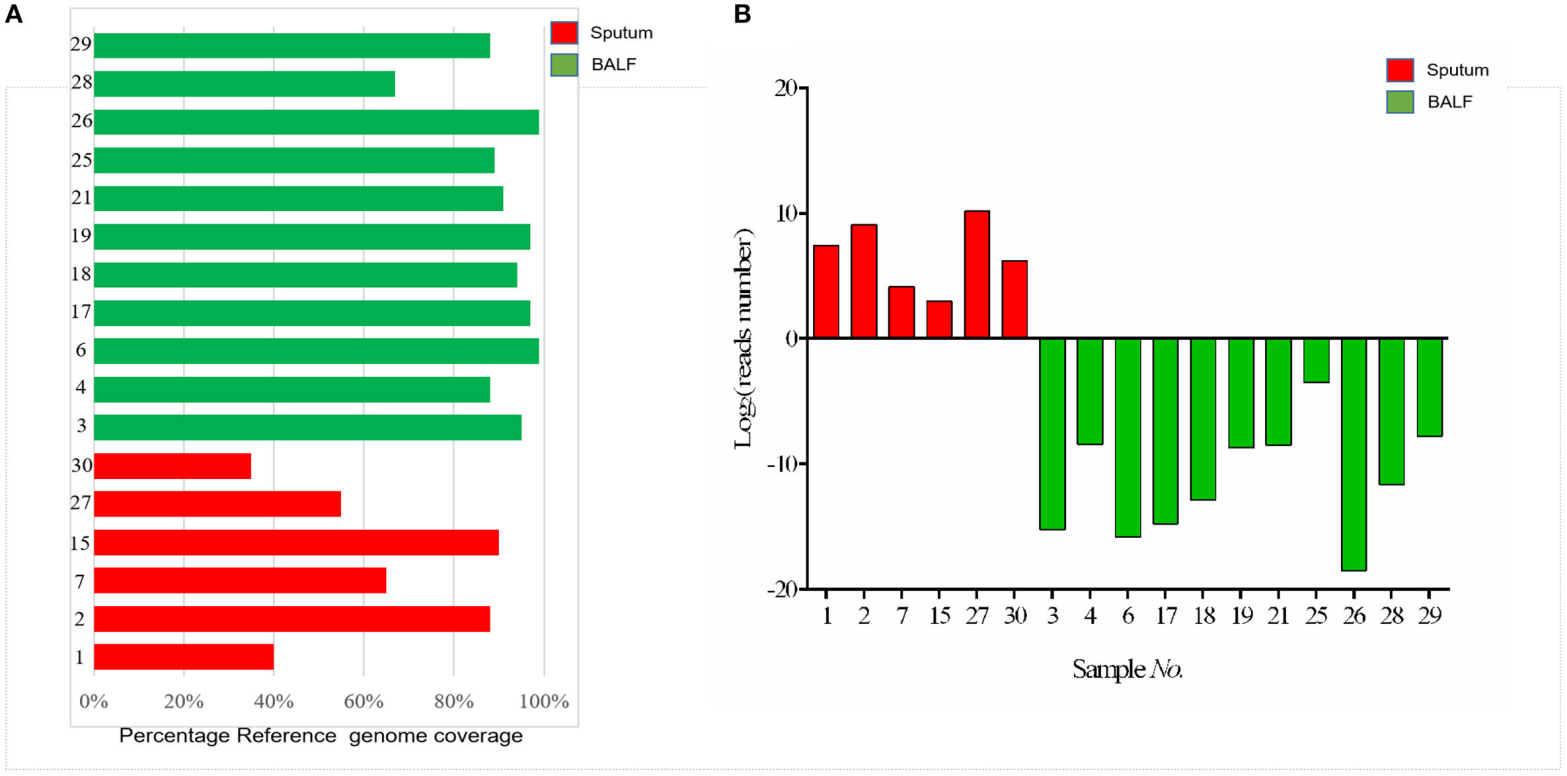
**(A)** Barplot showing the percentage reference genome coverage (a single read covering each genome position) for patients with sputum and BALF samples sequenced. **(B)** The number of mapped reads of *Mycobacterium tuberculosis* in each sample. The red columns represent the sputum samples; green columns represent the BALF samples.

The genomic coverage was analyzed in these 17 samples, and we found that five samples with DST-negative results had relatively low coverage (<85%) against the reference genome (Figure 4A). Among these five samples, it was noteworthy that four were sputum samples. In addition, to compare the effect of different sample types on sequencing results, the total number of reads of each sample mapped to the reference TB genome was counted. As shown in Figure 4B, the number of TB reads of BALF samples was significantly more than sputum samples (*p* < 0.05). Drug resistance was mostly discovered in BALF samples. These results indicated that the BALF sample was more suitable for detecting drug resistance by targeted capture sequencing.

## Discussion

In this study, we performed targeted capture sequencing using DNA from sputum and BALF specimens from 30 patients with TB suspects. A previous study indicated that DNA directly sequenced from sputum showed significantly more within-sample diversity than that from MGIT culture (Nimmo et al., 2019). In addition, NGS directly from sputum can quickly generate a complete genetic drug resistance profile, faster than MGIT culture sequencing (Nimmo et al., 2017). Otherwise, BALF specimens have been applied widely in the detection of pulmonary pathogenic bacteria infection including MTB and NTM (Theron et al., 2013; Yamasaki et al., 2015; Liu et al., 2018). In our study, samples sequenced from BALF had a higher reference genome coverage as compared with those obtained from sputum, and this was correlated to the number of lineage-specific reads in BALF samples that was significantly more than in sputum samples, and the drug resistance was mostly discovered in BALF samples (Figure 4). These results indicated that the BALF sample might be more suitable for detecting MTB types and drug resistance by targeted capture sequencing, which are consistent with a previous study: Liu et al. (2018) found BALF has significantly higher sensitivity (63.4%) than sputum (43.5%) for MTB detection by culture and nucleic acid amplification test; To et al. (2018) discovered that the sensitivity and specificity of GeneXpert for TB diagnosis using BALF was 80 and 98% in sputum AFB smear negative patients, respectively. The main reason is that some patients were bacteriologically negative due to lack of sputum or false-negative due to poor sputum sample quality.

MTB and NTM genotypes were determined in our research by detection of phylogenies with core genomic single nucleotide variants, as the Total Genotyping Solution for TB (TGS-TB) (Sekizuka et al., 2015). For acceptance as a diagnostic tool to guide treatment of patients with MTB and NTM infection, the sequencing platforms and analytical tools employed must be robust and reliable. Therefore, in our study, we compared the targeted capture sequencing results with other conventional TB diagnosis approaches. As shown in Figure 2, the overall positive rate of targeted capture sequencing (83.3%) was higher than AFB, LJ culture, TB-DNA, TB-RNA, MGIT 960, and even T-SPOT. Targeted capture sequencing had a highest consistency with MGIT 960, with 91.3% sensitivity and 83.3% accuracy. It is remarkable that the positive rates of MTB and NTM infection identified by targeted capture sequencing were both higher than MGIT 960 (MTB: 53.3 vs. 40%; NTM: 50 vs. 36.7%). The low specificities of targeted capture sequencing may indicate the low sensitivity of MGIT 960 for true MTB and NTM diagnosis, rather than false positives in patients without infection. These results indicated that targeted capture sequencing could be used as a highly sensitive and accurate method to diagnose TB, as well as MGIT 960. A possible explanation for this low sensitivity of TB-DNA and TB-RNA assay could be as follows: (1) NTM strains are not in the detection range; (2) the presence of PCR inhibitors; (3) insufficient mycobacteria load and nucleic acid material in some specimens which are possibly caused by the severity of the infection. For the TB-RNA assay, especially the RNA degradation (Suthar et al., 2013; Marangu et al., 2017).

In this study, targeted capture sequencing was used to perform molecular typing of NTM (including 32 human pathogenic NTM strains). Otherwise, 15 patients were identified as infected with seven human pathogenic NTM strains. As Bonaiti et al. (2015) described, patients affected by NTM infection frequently present some co-infections, which co-infected with other different NTM species, MTB, and other microorganisms. Among the 15 NTM-infected patients in our study, seven were MTB/NTM co-affected in this study. The most common isolated mycobacteria were *M*. *mu*c*ogenicum* and *M. abscessus*, which belong to the rapid-growing mycobacteria. Individuals with advanced HIV disease are more susceptible to NTM (Nyamogoba et al., 2012), and in our study, the only one HIV-infected patient was found co-infected with *M*. *mu*c*ogenicum*. The aforementioned results indicated that targeted capture sequencing is a meaningful method for the diagnosis and treatment of NTM infection diseases in clinic. However, because of the limitations of panel design, other NTM species, such as *M. avium* subsp. *paratuberculosis* (MAP), involved in the pathogenesis of Crohn's disease and other human autoimmune disorders, were uncovered in our study (Cao et al., 2018). To find out the cause of clinical infection symptoms, nested PCR or ELISA would be performed to identify these NTM species in our future study.

In this study, the average TTD was 18.5 ± 7.6 days for MGIT 960 with the extremity from 6 to 38 days. The median was 41.5 days for LJ culture with extremes from 13 to 96 days. T-SPOT test results were available within 1 to 2 days. For TB-DNA and TB-RNA test, the results were available within 3 days. The median TTD for target capture sequencing was 6 days (5–7 days), which was significantly faster than MGIT 960 and LJ culture (*p* < 0.01). Although the detection TTD of target capture sequencing is longer than that of T-SPOT, TB-DNA, and TB-RNA, considering the coverage and accuracy of detection, target capture sequencing is a relatively good method for clinical diagnosis of MTB and NTM.

Also, determining resistance to anti-TB drugs by targeted capture sequencing has been demonstrated as feasible and is being implemented in some specialist countries (Witney et al., 2015). Researchers observed high diagnostic accuracy for the detection of full drug resistance profile compared with standard DST testing performed in MGIT (Hasan et al., 2013). As an exhaustive library (1,325 mutations) predictive of DR for 15 anti-tuberculosis drugs was compiled (Coll et al., 2015), targeted capture sequencing is possible to determine DR. Our results show that the BACTEC MGIT 960 was reliable and highly sensitive for DST, as also shown by previous studies (Kim et al., 2013). The automated system was able to detect 18 XDR and 1 MDR isolates, whereas the targeted capture sequencing detected only nine drug resistance strains, which was inconsistent with other previous studies (10, 30, 33). Using targeted capture sequencing, only 22 of 1,325 drug resistance loci were discovered; mutations mainly occurred in *rrs* genes, and may lead to resistance to STR, CAP, KAN, and AMK.

The majority of the discordances seen here, however, were phenotypically resistant, with no resistance mutations found by genotypic methods. The discordances may be explained as follows: (1) These discordances are unlikely the result of errors in NGS but rather reflect the true absence of a mutated genotype within the genetic regions examined, especially the resistance loci of NTM may not be included in this mutation library, which is consistent with previous studies (Brown et al., 2015). (2) It is worth noting that MTB sequencing has mainly been performed from cultures and sequencing directly from clinical specimens such as sputum still needs to be optimized, and without the enrichment of MTB may not match the quality and quantity of data obtained via sequencing from clinical specimens (Witney et al., 2015). As seen in Figure 4, five samples with genomic coverage <85% failed to identify the drug resistance, and the number of reads was generally less in the 30 clinical samples, especially in sputum, which may result in false negative; (3) otherwise, the DST assay with MGIT 960 may show incidents of false-positive drug resistance (Chedore et al., 2010; Piersimoni et al., 2013; Colman et al., 2016). (4) Samples maybe deemed failures based on commonly used SNP calling thresholds employed by others in the field (Brown et al., 2015). Further work will be required to robustly establish and optimize parameters that are sufficient for clinical sample sequencing and interpretation, particularly when considering low-frequency variants. In addition, other DST methods, like Xpert MTB/RIF and LPA, should be performed to further verify the drug susceptibility results.

## Conclusion

We have constructed a targeted capture sequencing approach that provides rapid analysis of genome sequence data to describe the lineage of the MTB and NTM strains and predict resistance to 11 anti-TB drugs. This test was applicable to comprehensive and accurate diagnosis of MTB and NTM by comparing with other conventional phenotypic and molecular diagnostic approaches. However, from data presented here, the DST results identified by NGS showed a relative low consistency compared with MGIT 960. Further work should be done to verify the DST results and optimize the NGS parameters.

## Data Availability Statement

The datasets generated for this study can be found in the figshare repository, the link are as follows: https://figshare.com/s/ac0adbcc0b8caa9e3014; https://figshare.com/s/0f6a408d20d6b7b4f3e1.

## Ethics Statement

The studies involving human participants were reviewed and approved by the Ethics Committee of Shanghai Pulmonary Hospital (protocol no: 201709002). The patients/participants provided their written informed consent to participate in this study. Written informed consent was obtained from the individual(s) for the publication of any potentially identifiable images or data included in this article.

## Author Contributions

ZZ and DZ designed the experiments. ZZ and YH were involved in project administration. YH and XZ wrote and edited the article. ZG, DZ, and XZ performed experiments and analyzed the data. All authors have read and approved the final article.

## Conflict of Interest

XZ, ZG, and DZ were employed by Shanghai Yunying Medical Technology Co., Ltd. ZG, XZ, and DZ were employed by Jiaxing Yunying Medical Inspection Co., Ltd. The authors declare that this study received funding from Shanghai Yunying Medical Technology Co., Ltd. The funder had the following involvement in the study: designed the experiments, wrote and edited the manuscript, performed experiments and analyzed the data.
