# Supplementary material for: Comprehensive Determination of Mycobacterium tuberculosis and Nontuberculous Mycobacteria From Targeted Capture Sequencing
Source: Front Cell Infect Microbiol. 2020 Sep 1;10:449. doi: 10.3389/fcimb.2020.00449 (PMC7491257; doi:10.3389/fcimb.2020.00449)
Supplement: Supplementary file 1 [file Data_Sheet_1.docx]

Comprehensive determination of Mycobacterium tuberculosis and nontuberculous mycobacteria from targeted capture sequencing

He Y^1^, Gong ZY^2^, Zhao XK^2^, Zhang DY^2△^, Zhang ZS^1*^

Table S1. List of *mycobacterium tuberculosis* and *nontuberculous mycobacteria* strains

| ***Mycobacterium tuberculosis*** |
| --- |
| ***Mycobacterium africanum*** |
| ***Mycobacterium bovis*** |
| ***Mycobacterium microti*** |
| ***Mycobacterium tuberculosis*** |
| ***Nontuberculous mycobacteria*** |
| ***Mycobacterium abscessus*** |
| ***Mycobacterium aurum*** |
| ***Mycobacterium avium*** |
| ***Mycobacterium bohemicum*** |
| ***Mycobacterium brumae*** |
| ***Mycobacterium chelonae*** |
| ***Mycobacterium chubuense*** |
| ***Mycobacterium fortuitum*** |
| ***Mycobacterium gastri*** |
| ***Mycobacterium genavense*** |
| ***Mycobacterium gordonae*** |
| ***Mycobacterium haemophilum*** |
| ***Mycobacterium interjectum*** |
| ***Mycobacterium intracellulare*** |
| ***Mycobacterium kansasii*** |
| ***Mycobacterium lentiflavum*** |
| ***Mycobacterium mageritense*** |
| ***Mycobacterium marinum*** |
| ***Mycobacterium mucogenicum*** |
| ***Mycobacterium neoaurum*** |
| ***Mycobacterium nonchromogenicum*** |
| ***Mycobacterium peregrinum*** |
| ***Mycobacterium phlei*** |
| ***Mycobacterium scrofulaceum*** |
| ***Mycobacterium simiae*** |
| ***Mycobacterium smegmatis*** |
| ***Mycobacterium szulgai*** |
| ***Mycobacterium thermoresistibile*** |
| ***Mycobacterium triplex*** |
| ***Mycobacterium ulcerans*** |
| ***Mycobacterium vaccae*** |
| ***Mycobacterium xenopi*** |

Table S2. Summary of mutations included in the curated drug resistance library

| **Drug** | **Loci** | **No. variable sites** | **SNPs** | **Indels** |
| --- | --- | --- | --- | --- |
| INH | *katG* | 241 | 286 | 25 |
|  | *katG* promoter | 3 | 3 | 0 |
|  | *inhA* | 12 | 15 | 0 |
|  | *inhA* promoter | 9 | 11 | 0 |
|  | *ahpC* | 8 | 8 | 0 |
|  | *ahpC* promoter | 13 | 14 | 0 |
|  | *kasA* | 8 | 11 | 0 |
| RMP | *rpoB* | 89 | 135 | 19 |
|  | *rpoC* | 8 | 8 | 0 |
| EMB | *embB* | 123 | 153 | 1 |
|  | *embA* | 5 | 5 | 0 |
|  | *embA* promoter | 3 | 3 | 0 |
|  | *embC* | 25 | 26 | 0 |
|  | *embR* | 22 | 24 | 0 |
| STR | rrs | 21 | 25 | 0 |
|  | rpsL | 14 | 19 | 0 |
| PZA | *pncA* | 215 | 269 | 64 |
|  | *pncA* promoter | 4 | 6 | 0 |
|  | *rpsA* | 3 | 4 | 0 |
|  | *panD* | 9 | 11 | 1 |
| ETH | *ethA* | 33 | 29 | 5 |
|  | *ethR* | 3 | 4 | 0 |
|  | *inhA* promoter | 3 | 3 | 0 |
|  | *inhA* | 3 | 3 | 0 |
| FLQs | *gyrA* | 15 | 22 | 0 |
|  | *gyrB* | 22 | 29 | 0 |
| AMK | rrs | 8 | 9 | 0 |
| CAP | rrs | 3 | 4 | 0 |
|  | tlyA | 26 | 18 | 10 |
| KAN | rrs | 3 | 4 | 0 |
|  | eis promoter | 9 | 10 | 0 |
| PAS | thyA | 23 | 17 | 5 |
|  | folC | 16 | 19 | 0 |
|  | ribB | 1 | 1 | 0 |
| LZD | rrl | 2 | 2 | 0 |
|  | rplC | 1 | 1 | 0 |
| BDQ CFZ | Rv0678 | 7 | 5 | 2 |

Table S3. The clinical informations and targeted capture sequencing diagnosis results of 30 enrolled patients

| **Patient No.** | **Gender** | **Age** | **TB** | **NTM Typing** |
| --- | --- | --- | --- | --- |
| **1** | female | 68 | + | *M. abscessus* |
| **2** | male | 59 | + | *-* |
| **3** | male | 64 | + | *-* |
| **4** | male | 28 | + | *M. abscessus*  *M. mucogenicum* |
| **5** | female | 69 | - | *M. abscessus* |
| **6** | male | 32 | + | *M. mucogenicum* |
| **7** | male | 24 | + | *-* |
| **8** | female | 66 | - | *M. mucogenicum* |
| **9** | male | 69 | - | *-* |
| **10** | female | 70 | - | *-* |
| **11** | male | 64 | - | *-* |
| **12** | female | 80 | - | *M. abscessus* |
| **13** | female | 30 | - | *M. mucogenicum* |
| **14** | female | 75 | - | *-* |
| **15** | male | 59 | + | *-* |
| **16** | male | 65 | - | *-* |
| **17** | male | 25 | + | *-* |
| **18** | female | 29 | + | *-* |
| **19** | male | 67 | + | *M. avium、*  *M. cellulare、M. mucogenicum* |
| **20** | female | 20 | - | *M. chelonae* |
| **21** | male | 60 | + | *M. chelonae* |
| **22** | female | 57 | - | *M. mucogenicum* |
| **23** | female | 68 | - | *M. fortuitum* |
| **24** | female | 52 | - | *M. mucogenicum* |
| **25** | male | 54 | + | *M. xenopi* |
| **26** | male | 47 | + | *-* |
| **27** | male | 20 | + | *-* |
| **28** | male | 62 | + | *M. avium*  *M. intracellulare* |
| **29** | male | 81 | + | *-* |
| **30** | male | 49 | + | *-* |

Table S4. The MTB and NTM diagnosis results of 30 enrolled patients

| **Patient No.** | **AFB** | **LJ culture** | **TB-DNA** | **TB-RNA** | **T-spot** | **BACTEC MGIT 960** | **targeted capture sequencing** |
| --- | --- | --- | --- | --- | --- | --- | --- |
| **1** | + | + | - | - | + | + | + |
| **2** | + | + | - | - | + | + | + |
| **3** | + | + | + | + | + | + | + |
| **4** | + | + | + | + | + | + | + |
| **5** | - | + | - | - | - | + | + |
| **6** | + | + | + | + | + | + | + |
| **7** | - | - | - | + | + | + | + |
| **8** | - | - | - | - | + | + | + |
| **9** | + | - | - | - | - | + | - |
| **10** | - | - | - | - | - | - | - |
| **11** | - | - | + | + | + | - | - |
| **12** | + | + | - | - | - | + | + |
| **13** | - | - | - | - | - | - | + |
| **14** | - | - | + | - | + | - | - |
| **15** | - | - | - | - | + | + | + |
| **16** | + | - | + | - | + | + | - |
| **17** | + | + | + | + | + | + | + |
| **18** | - | - | - | - | + | - | + |
| **19** | + | + | + | + | + | + | + |
| **20** | - | - | - | - | + | + | + |
| **21** | + | + | + | + | + | + | + |
| **22** | - | - | - | - | - | + | + |
| **23** | + | + | - | - | - | + | + |
| **24** | + | - | + | - | + | + | + |
| **25** | + | - | - | - | + | + | + |
| **26** | - | - | + | + | + | - | + |
| **27** | - | - | - | - | + | + | + |
| **28** | + | + | - | - | + | + | + |
| **29** | - | - | + | - | + | - | + |
| **30** | - | - | - | - | + | + | + |
